# Impact on urinary oxalate levels with use of ezetimibe

**DOI:** 10.1002/edm2.221

**Published:** 2021-01-04

**Authors:** Christopher L. Trautman, Margaret Van Cleve, Emily A. Sullivan, Colleen T. Ball, Jordan J. Cochuyt, Ivan Porter

**Affiliations:** ^1^ Division of Nephrology and Hypertension Mayo Clinic Florida Jacksonville FL USA; ^2^ CRISP Mayo Clinic Florida Jacksonville FL USA; ^3^ Division of Biomedical Statistics and Informatics Mayo Clinic Florida Jacksonville FL USA

## Abstract

**Background:**

Calcium oxalate stones are the most common cause of nephrolithiasis in the United States. Smaller studies of <15 patients investigating ezetimibe, a selective cholesterol absorption inhibitor, have suggested increased urine oxalate levels with use of the drug. We attempt to better define this relationship of ezetimibe on urinary oxalate using a larger patient sample analysing multiple urine collections on and off treatment.

**Materials and Methods:**

We retrospectively reviewed all consecutive patients from 01/2018 through 04/2019 evaluated for nephrolithiasis with use of ezetimibe documented in their medical record at Mayo Clinic Florida. Primary outcomes included increase in urinary oxalate with use of ezetimibe and reduction in urinary oxalate with discontinuation of medication.

**Results:**

Of 57 reviewed patients, 30 (53%) met inclusion criteria yielding 117 24‐h urine measurements either on ezetimibe (72 measurements) or off ezetimibe (41 measurements). The mean urinary oxalate level off ezetimibe was 39.86 mg versus 40.45 mg with ezetimibe. After adjusting for age and sex, the estimated difference was 1.239 mg (95% CI, −4.856 to 7.335 mg; *p* = 0.93). A subset of six patients with urinary oxalate values both on and off ezetimibe showed a difference in 24‐h urinary oxalate levels ranged from −16.40 to 14.95 mg (mean difference = 0.93 mg; median difference = 3.84 mg).

**Conclusion:**

Use of ezetimibe does not provide clear evidence of a difference in urinary oxalate levels.

## Graphical abstract

We attempt to better define this relationship of ezetimibe on urinary oxalate using a larger patient sample analyzing multiple urine collections on and off treatment. Use of ezetimibe does not provide clear evidence of a difference in urinary oxalate levels.

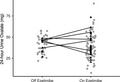

## 1 INTRODUCTION

Kidney stones are common in the United States with an estimated prevalence of 10.6% in men and 7.1% in women.^1^ Nearly 75% of analysed stones are comprised of calcium oxalate with or without trace amounts of secondary minerals such as uric acid or calcium phosphate.^2^ Signs and symptoms of nephrolithiasis range from asymptomatic incidental findings on imaging tests to acute renal colic with haematuria, nausea, vomiting and hydronephrosis potentially necessitating urgent urologic intervention. Risk factors for calcium oxalate stone formation include dietary intake of oxalate, sodium, and ascorbic acid, anatomic variants, urine volume plus composition, and other medical conditions such as primary hyperthyroidism, obesity and gastrointestinal disorders.^3^

Enteric hyperoxaluria results from fat malabsorption syndromes of the intestinal tract allowing free fatty acids to bind dietary calcium instead of dietary oxalate.^4^ Soluble oxalate readily moves into the bloodstream which becomes filtered by the kidneys inducing higher levels of urinary oxalate. Conditions such as inflammatory bowel disease and bariatric surgery with bowel resection can precipitate enteric hyperoxaluria along with drugs such as orlistat, an oral lipase inhibitor indicated for weight loss via reducing absorption of dietary fat.^5^ In 2004, the Food and Drug Administration (FDA) approved the first selective cholesterol absorption inhibitor, ezetimibe, for treatment of hyperlipidemia.^6^ This drug acts on intestinal lumen limiting the transport of biliary and dietary cholesterol into enterocytes and reducing chylomicron synthesis causing reductions in serum low‐density lipoprotein (LDL) levels.^7^ Subsequently, this increases the concentration of free cholesterol in the intestinal tract which may impact oxalate absorption. Previous analysis in a small number of cases has suggested increased urine oxalate levels with use of ezetimibe. In the present study, we examined a relationship of ezetimibe and urinary oxalate, in patients with urinary stone disease, analysing results of multiple urine collections while on and off treatment with ezetimibe.

## 2 MATERIALS AND METHODS

Institutional review board (IRB) approval was obtained for this study. We retrospectively reviewed all consecutive patients from January 2018 through April 2019 evaluated for nephrolithiasis at a single nephrology clinic with use of ezetimibe documented in their medical record. Demographics were obtained through an internal electronic medical records system including age, gender and ethnicity. Laboratory studies including serum creatinine, uric acid, LDL, vitamin D 25‐OH levels and urine supersaturation profiles including 24 h urine oxalate were analysed according to a standard protocol.^8^ Estimated glomerular filtration rate was calculated using the 2009 Chronic Kidney Disease Epidemiology Collaboration (CKD‐EPI) equation.^9^ Inclusion criteria consisted of age ≥18 years, lipid treatment with ezetimibe alone or in combination with a statin and at least one 24 h urine oxalate measurement. Patient's meeting inclusion criteria had all of their 24 h urine collections analysed including samples while off ezetimibe for comparison.

### 2.1 Statistical analysis

Means and standard deviations were calculated for continuous variables, counts and percentages for categorical data. Urinary oxalate values were calculated using the arithmetic mean for those who had more than one. Dot plots were constructed to graphically display urinary oxalate values while on and off ezetimibe with lines connecting the values of those patients with urinary oxalate values both on and off ezetimibe. Patient‐averaged urinary oxalate values were calculated using the arithmetic mean for those who had more than one, separately while on ezetimibe and while off ezetimibe. For the primary aim of exploring the relationship between ezetimibe use and urinary oxalate levels, we utilized unadjusted and age‐ and sex‐adjusted generalized estimating equations to account for the repeated measurements of urinary oxalate within patient. In an additional analysis, we summarized the differences in urinary oxalate levels with versus without ezetimibe use in the subset of patients who had urinary oxalate levels under both conditions. All statistical tests were two‐sided. Statistical analyses and graphics were performed using SAS (version 9.4; SAS Institute Inc.).

## 3 RESULTS

A total of 57 patients were reviewed over the 16 month time interval with 30 (53%) patients meeting inclusion criteria. Most common reasons for exclusion from analysis were incomplete urine supersaturation profile or non‐collection of urine despite recommendation and electronic order during nephrology clinic visit. Patient characteristics (Table 1) demonstrate that 23 patients (77%) were male, average age was 68.8 years, and most common type of documented stone was calcium oxalate (41%). Of note, not all patients had available serum LDL and vitamin D 25‐OH levels. Only one patient included in the analysis had a history of gastric bypass.

**TABLE 1:** Patient characteristics

Variable	Summary (*N* = 30)
Age, mean (SD), years	68.8 (8.9)
Male sex, no. (%)	23 (77%)
Race, no. (%)	
African American	3 (10%)
Asian	1 (3%)
White	24 (80%)
Chose not to disclose	2 (7%)
GFR >60 ml/min, no. (%)	22 (73%)
Serum uric acid, mean (SD), mg/dl	5.9 (1.3)
LDL, mean (SD), mg/dl	85.7 (28.9)
Vitamin D−25 OH, mean (SD), ng/ml	34.4 (11.7)
History of bariatric surgery	1 (3%)
History of inflammatory bowel disease	0 (0%)
Stone type	
Calcium oxalate	7 (41.2%)
Mixed calcium	5 (29.4%)
Mixed calcium oxalate / uric acid	2 (11.8%)
Uric acid	3 (17.6%)

Note

Information was not available for the following variables: LDL (*n* = 9), vitamin D‐25 OH (*n* = 11), and stone type (*n* = 13).

**FIGURE 1:**
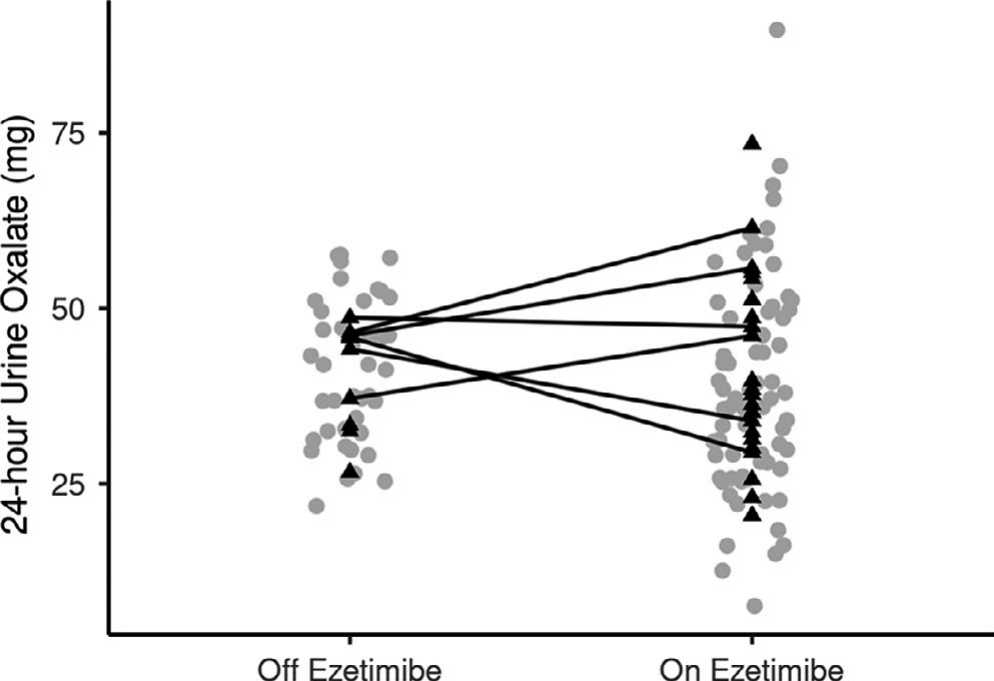
Urinary oxalate levels according to ezetimibe use. Grey circles represent the 113 24‐h urine oxalate measurements (*N* = 28 patients), the patient‐averaged 24‐h urine oxalate measures are represented by black triangles, and connected lines represent the patient‐averaged 24‐h urine oxalate for the 6 patients who had measurements both on and off ezetimibe

Seventy‐two 24‐h urine oxalate levels were available for analysis in 23 patients during use, and 41 in 11 patients after discontinuing use of Ezetimibe. The mean urinary oxalate level was 39.86 mg while off ezetimibe and 40.45 mg while on ezetimibe. After adjusting for age and sex, the estimated difference was 1.239 mg (95% CI −4.856 to7.335 mg), *p* = 0.69) (Table 2). In the subset of six patients who had urinary oxalate values both on and off ezetimibe, the difference in 24‐h urinary oxalate levels ranged from −16.40 to 14.95 mg (mean difference = 0.93 mg; median difference = 3.84 mg). Three of the six patients had a decrease in 24‐hr urinary oxalate levels with ezetimibe use (Figure 1).

**TABLE 2:** Differences in 24‐h urinary oxalate levels with ezetimibe use

Dependent variable	Difference in urinary oxalate (on ezetimibe vs. off ezetimibe)			
	Unadjusted difference in mean urinary oxalate (95% CI), on ezetimibe vs. off ezetimibe	P	Age‐ and sex‐adjusted difference in urinary oxalate (95% CI), on ezetimibe vs. off ezetimibe	P
Urinary oxalate, mg	0.025 (−6.289 to 6.338)	0.99	1.239 (−4.856 to 7.335)	0.69

Note

The differences in mean urinary oxalate levels were estimated from single generalized estimating equations to account for the repeated measurement of urinary oxalate levels within patient.

Abbreviation: CI, confidence interval.

## 4 DISCUSSION

Urinary oxalate is a key factor in the development of kidney stones, a common malady, and has been shown to be increased in the setting of fat malabsorption. Ezetimibe is a cholesterol absorption inhibitor and is widely used as a lipid lowering compound. Preliminary findings suggesting a link between ezetimibe use and increased urinary oxalate levels led to the present study examining a relationship between ezetimibe and urinary oxalate in patients with urinary stone disease, while analysing multiple urine collections. We found no evidence of effect of ezetimibe on urinary oxalate levels. We are not aware of any other studies addressing this question.

Urinary oxalate levels are impacted by gastrointestinal disorders and intra‐luminal medications such as orlistat and cholestyramine.^4^ Use of lipase inhibitors for weight loss prevents hydrolysis of triglycerides into free fatty acids thereby limiting intestinal absorption.^5^ Dietary calcium interacts with excess triglycerides inducing saponification leading to a reduction of free calcium to bind oxalate.^10^ By comparison, ezetimibe blocks uptake of luminal cholesterol resulting in increased amounts of free cholesterol in the small bowel without affecting triglycerides or fat soluble vitamins.^6^ Free cholesterol is eliminated in faeces by degradation into biliary salts or as neutral sterols which are unlikely to significantly influence oxalate absorption.^11^

This retrospective study had a number of limitations. This single centre study was limited by modest patient sample size. Furthermore, the study was not designed to determine cause and effect and did not control for confounding variables such as dietary changes. In these cases, the use of ezetimibe was inferred by chart review of medication list as medication compliance was not closely monitored. Patients were seen by different providers over time which may influence the effect of stone education via modifiable risk factors on future urine collections. An ideal prospective study design would allow for a baseline urinary profile prior to initiation of ezetimibe with robust and complete dietary review at regular intervals with subsequent urine collections.

In conclusion, this study provided no evidence of effect of ezetimibe use on urinary oxalate levels in patients with nephrolithiasis being evaluated in the nephrology clinic setting.

## CONFLICT OF INTEREST

The authors of this manuscript have no conflicts of interest to disclose. The data that support the findings of this study are available from the corresponding author upon reasonable request.

## AUTHOR CONTRIBUTIONS

Christopher L. Trautman, M.D. – concept & design, drafting article. Margaret Van Cleve – data collection. Emily A. Sullivan – data collection. Colleen T. Ball, M.S. – statistics, data interpretation. Jordan J. Cochuyt – statistics, data interpretation. Ivan E. Porter M.D. – concept & design, mentorship, drafting, revisions.

## Supporting information

Table S1
