# Supplementary material for: Impact on urinary oxalate levels with use of ezetimibe
Source: Endocrinol Diabetes Metab. 2021 Jan 4;4(2):e00221. doi: 10.1002/edm2.221 (PMC8029507; doi:10.1002/edm2.221)
Supplement: Supplementary file 1 — Table S1 [file EDM2-4-e00221-s001.docx]

**Supplemental Table 1: Average of 24 Hour Urinary Risk Profiles**

| Urinary Variable | On Ezetimibe (N=72) | Off Ezetimibe (N=41) |
| --- | --- | --- |
| Ammonium, mmol | 24 | 35 |
| Calcium, mg | 171 | 253 |
| Citrate, mg | 633 | 777 |
| Magnesium, mg | 103 | 121 |
| Oxalate, mg | 38.4 | 41.0 |
| Phosphorus, mg | 824 | 1142 |
| Sodium, mmol | 147 | 196 |
| Sulfate, mmol | 19 | 24 |
| Uric Acid, mg | 565 | 677 |
| Calcium Oxalate Crystal | 1.12 | 1.31 |
| Volume, mL | 2091 | 2098 |
| Creatinine, mg | 1465 | 1797 |
